# Development and validation of a nomogram for assessing hepatocellular carcinoma risk after SVR in hepatitis C patients with advanced fibrosis and cirrhosis

**DOI:** 10.1186/s13027-024-00578-3

**Published:** 2024-04-25

**Authors:** Shanshan Xu, Lixia Qiu, Liang Xu, Yali Liu, Jing Zhang

**Affiliations:** 1 The Third Unit, Department of Hepatology, Beijing Youan Hospital, Capital Medical University, Beijing, 100069 People’s Republic of China; 2 Department of Hepatology, Tianjin Second People’s Hospital, Tianjin Research Institute of Liver Diseases, Tianjin, 300192 People’s Republic of China

**Keywords:** Hepatitis C, Hepatocellular carcinoma, Sustained virological response, Nomogram

## Abstract

**Background:**

Hepatitis C patients with advanced fibrosis or cirrhosis are at high risk of developing hepatocellular carcinoma (HCC), even after sustained virological response (SVR). Clinical recommendations impose a significant burden on patients by recommending lifelong screening for HCC every six months. The goals of this study were to develop a nomogram that accurately stratifies risk of HCC and improve the screening approach that is currently in use.

**Method:**

Risk factors for HCC were identified using univariate and multivariate analyses in this prospective study. We developed and validated a nomogram for assessing hepatocellular carcinoma risk after SVR in patients with advanced fibrosis and cirrhosis.

**Results:**

During the median follow-up period of 61.00 (57.00–66.00) months in the derivation cohort, 37 patients (9.61%) developed HCC. Older age (HR = 1.08, 95% CI 1.02–1.14, *p* = 0.009), male gender (HR = 2.38, 95% CI 1.10–5.13, *p* = 0.027), low serum albumin levels (HR = 0.92, 95% CI 0.86–1.00, *p* = 0.037), and high liver stiffness measurement (LSM) (HR = 1.03, 95% CI 1.01–1.06, *p* = 0.001) were found to be independent predictors of HCC development. Harrell's C-index for the derivation cohort was 0.81. The nomogram’s 3-, 5- and 7-years time-dependent AUROCSs were 0.84 (95% CI 0.80–0.88), 0.83 (95% CI 0.79–0.87), and 0.81 (95% CI 0.77–0.85), respectively (all *p* > 0.05). According to the nomogram, patients are categorized as having low, intermediate, or high risk. The annual incidence rates of HCC in the three groups were 0.18%, 1.29%, and 4.45%, respectively (all *p* < 0.05).

**Conclusions:**

Older age, male gender, low serum albumin levels, and high LSM were risk factors for HCC after SVR in hepatitis C patients with advanced fibrosis and cirrhosis. We used these risk factors to establish a nomogram. The nomogram can identify a suitable screening plan by classifying hepatitis C patients according to their risk of HCC.

**Supplementary Information:**

The online version contains supplementary material available at 10.1186/s13027-024-00578-3.

## Introduction

Hepatitis C virus (HCV) is widespread globally, and all individuals, regardless of race, sex, or age, are susceptible to this virus. At the beginning of 2020, there were an estimated 56,800,000 viraemic HCV infections globally [1]. World Health Organization estimates that each year 399,000 people die from HCV-related cirrhosis and hepatocellular carcinoma (HCC) [2]. The incidence rate of HCC is between 1 and 3% after 30 years of HCV infection, especially in patients with advanced fibrosis and cirrhosis [3]. With the widespread use of direct-acting antiviral agents (DAAs), the majority of patients are cured, and the risk of HCC is decreased by almost 70% [4–8]. However, many studies have confirmed that individuals with advanced fibrosis or cirrhosis are still at high risk of HCC and should be checked for this disease every six months for the remainder of their lives [9–11]. AASLD and EASL guidelines recommend this policy, which imposes a heavy burden on patients. Therefore, to improve the screening strategy, better risk assessment is urgently needed.

Given the importance of early detection and diagnosis of HCC in enhancing long-term prognosis, researchers have made great efforts in recent years to develop prediction models, risk calculators, biomarkers, genetic panels and in-depth learning models [12]. Most of these tools were developed based on a hepatitis B cohort and not validated in chronic hepatitis C patients. Indeed, few HCC risk models have been created specifically for patients with a sustained virological response (SVR) to hepatitis C virus (HCV). Ioannou [13] developed a set of models in 2018 using a sizable retrospective cohort that included 45,810 patients from the National Health Service of the Veterans Affairs. Based on cirrhosis status and hepatitis C virus levels, four formulas were included in the models, each of which was appropriate for a different set of circumstances. In addition to being complicated, the model lacked validation. Azzi [14] developed a model that focused on advanced fibrosis patients who received DAA therapy and achieved SVR. The model was based on the ANRS CO22 HEPATHER cohort. The AUROC of the validation cohort was 0.61, and that of the derivation cohort was 0.76. Additionally, several studies have assessed the risk of HCC using transient elastography [15–17] or the FIB-4 score [17, 18]. Moreover, none of these models have been generally accepted or corroborated by additional research.

In this study, we aimed to develop a nomogram based on a prospective cohort of hepatitis C patients who achieved SVR to improve the effectiveness of the HCC screening approach because prior models for hepatitis C patients are either too complex or have very low predictive ability.

### Patients and methods

Patients in our study cohort were residents of a county in Hebei Province, China, and became infected with HCV through blood transfusions in the 1990s. In total, 2892 patients were treated with peg-interferon ± ribavirin (PR) and/or direct-acting antiviral agents (DAAs) in our hospital beginning in 2015. The inclusion criteria: (1) age ≥ 18, no gender limitation; (2) all patients acheived SVR; (3) the initial diagnosis of cirrhosis or severe fibrosis was made. The exclusion criteria were as follows: (1) combined with chronic hepatitis B; (2) HCC diagnosed within half a year after SVR; (3) other system malignant tumors; and (4) HIV antibody positive. In total, 551 hepatitis C patients with baseline advanced fibrosis or cirrhosis were included in our study. Patients were monitored at six-month intervals until 24 May 2022 or until development of HCC.

External validation of the model was performed in a cohort of 211 hepatitis C patients from Tianjin Second People's Hospital in China. All patients received DAA therapy and achieved SVR. The inclusion and exclusion criteria used were similar to those used in our study. Patients were followed up every three to six months until April 2020 or HCC development.

### Standard care and data collection

The patients were treated according to established guidelines and medication instructions, by providing a PR or DAA or by shifting to the DAA if the PR failed (PR + DAA). SVR was defined as 12 weeks after DAA therapy and 24 weeks after PR therapy. The start of treatment was referred to as the baseline.

Clinical data, including demographic data, anthropometric indices, past medical history, liver function, coagulation function, routine blood test results, HCV load and genotype, alpha-fetoprotein (AFP), imaging and pathological examination, etc., were collected at baseline. Liver function, coagulation function, routine blood test results, AFP, and other imaging indices were collected at each follow-up visit. Liver stiffness measurement (LSM) was measured by FibroScan® 502 (Echosens, France). An LSM ≥ 10 kPa was defined as advanced fibrosis. Cirrhosis was diagnosed according to laboratory results and imaging, an LSM > 13 kPa, or a FIB-4 > 3.25 [9]. FIB-4 = (Age × AST)/(platelet count × $\surd {\text{ALT}})$. Delta-LSM and delta-FIB-4 were defined as the differences between baseline and 12–24 weeks after SVR. HCC was diagnosed by histopathology or by one-two imaging methods (enhanced CT, MRI, or contrast-enhanced ultrasound) [19]. The definition of alcohol use disorder was 140 g/w for women and 210 g/w for men.

### Statistical analysis

Statistical analysis was performed using the Statistical Package for the Social Sciences (SPSS version 23.0) and R (version 4.2.1). Variables are expressed as counts and percentages for categorical variables and as medians and interquartile ranges (IQRs) for continuous variables. Categorical variables were compared using the chi-square test or Fisher’s exact test, and continuous variables were compared by Student’s t test or the Mann‒Whitney U test, when appropriate.

Univariate and multivariate Cox proportional hazards regression models were used to estimate the effect of variables on the risk of HCC occurrence and to develop the HCC prediction model. A time-dependent receiver operating characteristic (ROC) curve was used to evaluate the prediction accuracy of the model. The model's discriminant performance was assessed using Harrell's C-index. The concordance between the observed probability and the HCC probability predicted by the model was graphically assessed using a calibration map.

The HCC risk score was determined using the hazard ratio of the predictive variables. The cumulative incidence of HCC among groups of patients with different risk scores was evaluated via Kaplan‒Meier curves and compared via the log-rank test.

Finally, the model’s prediction accuracy was assessed using receiver operating characteristic (ROC) curves. Comparison between pairs of models was performed using the method of Delong et al.

## Results

### Patient characteristics

**Fig. 1:**
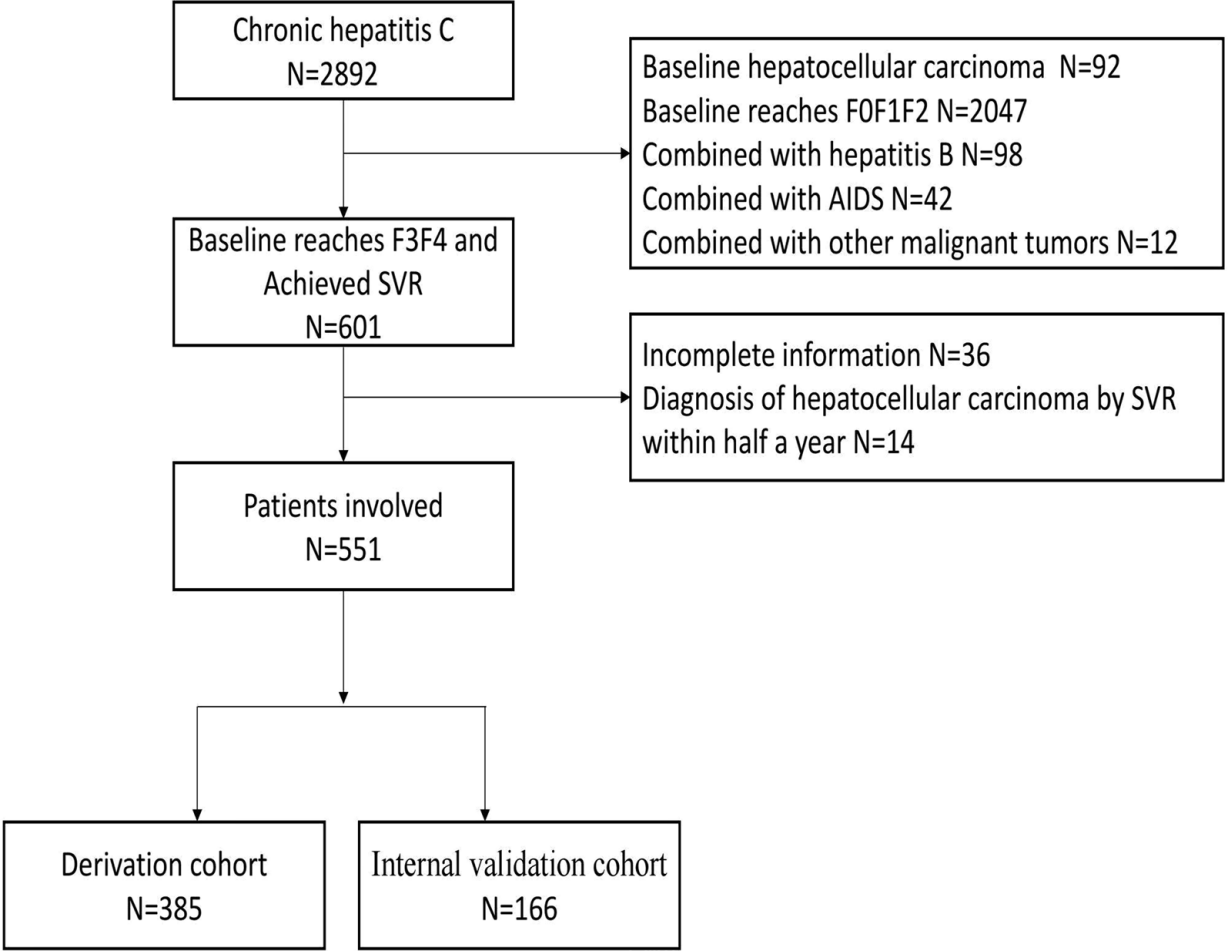
Study cohort inclusion and exclusion flow chart with outcome

A total of 551 adult hepatitis C patients with baseline advanced fibrosis or cirrhosis after SVR were included in this study. The first patient was recruited on 8 January 2015, and the last visit was on 24 May 2022. During the course of the median follow-up period of 69.9 (60.7–74.9) months, 53 patients (9.62%) developed HCC. The patients were randomly divided into a derivation cohort (70%, n = 385) and an internal validation cohort (30%, n = 166). Figure 1 displays the flow chart. Table 1 lists all the patients' baseline indicators as well as those of the patients with HCC.

**Table 1:** Baseline characteristics of patients

	Derivation cohort		Internal validation cohort		External validation cohort		*p1* Value	*p2* Value
	All patients (n = 385)	HCC (n = 37)	All patients (n = 166)	HCC (n = 16)	All patients (n = 211)	HCC (n = 20)		
Age—years	61.00(57.00–66.00)	64.00(59.00–67.00)	60.50(57.00–66.00)	64.50(60.00–69.00)	57.00(50.00–62.00)	61.00(58.00–67.00)	0.671	** < 0.001**
Male (%)	44.42	64.86	39.16	68.75	38.86	35.00	0.252	0.190
Follow-up time (months)	66.60 ± 12.30	47.30(30.40–58.80)	65.20 ± 13.90	37.20(28.10–51.60)	27.80 ± 13.50	26.80 ± 18.10	0.226	** < 0.001**
BMI (kg/m^2^)	26.74 ± 3.83	25.45(22.83–27.62)	26.87 ± 9.28	25.00(24.24–26.23)	24.89 ± 4.69	26.39 ± 3.14	0.872	** < 0.001**
Hypertension (%)	36.36	24.32	37.95	43.75	24.64	20.00	0.092	**0.015**
Diabetes (%)	24.42	27.03	31.33	12.50	23.70	35.00	0.950	0.845
Hyperlipidemia (%)	12.73	5.41	20.48	18.75	–	**–**	**0.020**	–
Alcohol use disorder (%)	13.77	27.03	16.27	31.25	25.59	25.00	0.445	** < 0.001**
*Fibrotic stage (%)*								
≥ F3	83.64	70.27	89.76	75.00	84.83	75.00	0.061	0.702
Decompensated cirrhosis	16.36	29.73	10.24	25.00	15.17	25.00	0.061	0.702
*Antiviral regimen (%)*								
PEG ± RBV	37.92	21.62	31.93	6.25			0.179	–
DAA	45.45	62.16	47.59	56.25	74.41	50.00	0.644	** < 0.001**
PR + DAA	16.63	16.22	20.48	37.50	25.59	50.00	0.277	**0.009**
*HCV genotype (%)*								
1b	64.94	70.27	69.28	93.75	63.51	75.00	0.323	0.643
2a	27.79	18.92	22.89	–	21.80	20.00	0.231	0.109
3a	–	–	–	–	3.32	–	–	–
3b	–	–	–	–	4.26	5.00	–	–
6a	–	–	–	–	7.11	–	–	–
Undetected	7.27	10.81	7.83	6.25	–	–	0.819	** < 0.001**
HCV-RNA (log IU/ml)	6.00 ± 0.87	5.90(5.02–6.66)	6.14 ± 0.85	6.35(5.56–6.85)	5.59 ± 0.84	5.63 ± 0.81	0.183	** < 0.001**
WBC (× 10^9^/L)	4.89(3.73–6.02)	4.09(2.96–5.08)	4.98(3.85–6.40)	4.92(3.19–6.25)	4.19(3.13–5.45)	3.37(2.94–4.85)	0.625	** < 0.001**
HGB (g/L)	137.56 ± 20.11	125.29 ± 24.32	136.06 ± 17.59	132.80(113.25–158.00)	132.77 ± 22.92	126.50(107.00–139.00)	0.471	0.074
Platelet count (× 10^9^/L)	132.00(89.00–178.00)	108.73 ± 69.88	119.50(79.25–167.25)	80.50(62.00–121.75)	89(65.00–142.00)	76.50(40.50–97.00)	0.196	** < 0.001**
ALT (IU/L)	77.46 ± 61.98	79.35 ± 49.70	71.09 ± 53.81	68.50(45.50–86.75)	67.73 ± 50.66	45.85 ± 26.97	0.302	**0.040**
AST (IU/L)	70.39 ± 45.49	81.91 ± 39.22	67.32 ± 40.63	59.00(41.75–93.75)	68.84 ± 44.24	74.54 ± 48.12	0.556	0.809
GGT (IU/L)	57.43 ± 46.77	60.30 ± 45.08	58.50 ± 58.33	79.20 ± 91.58	105.69 ± 186.37	82.54 ± 85.93	0.807	**0.001**
Albumin (g/L)	43.00(39.70–45.10)	39.55(34.55–42.70)	42.55(39.30–44.58)	42.20(37.18–44.75)	40.10(36.10–43.50)	37.10(32.55–39.10)	0.880	** < 0.001**
Bilirubin (µmol/L)	17.50(13.80–23.40)	27.09 ± 17.22	17.95(14.00–25.98)	20.70(14.25–27.18)	18.00(14.10–23.50)	26.85(16.25–40.95)	0.619	** < 0.001**
Cr (µmol/L)	52.10(45.40–60.40)	54.10(47.95–63.98)	53.00(45.42–58.70)	62.41 ± 30.12	56.50(44.50–61.50)	57.00(37.00–72.00)	0.609	**0.001**
INR	1.05(0.99–1.14)	1.18 ± 0.21	1.05(0.99–1.14)	1.03(0.99–1.14)	1.10(1.01–1.22)	1.14(1.06–1.30)	0.523	** < 0.001**
AFP (ng/mL)	12.32 ± 20.47	16.14 ± 17.15	13.40 ± 22.16	21.57 ± 46.30	17.66 ± 22.70	15.71 ± 12.88	0.608	**0.001**
CTP	5.34 ± 0.89	5.97 ± 1.43	5.41 ± 1.02	5.50 ± 1.27	5.46 ± 1.01	6.31 ± 1.84	0.083	0.203
LSM (kPa)	14.9(10.80–20.90)	26.55 ± 15.10	15.10(10.80–20.48)	22.00(13.65–40.75)	21.3(14.00–34.30)	33.65(23.08–66.95)	0.566	** < 0.001**
Delta-LSM (kPa)	6.09 ± 8.07	8.83 ± 11.96	5.42 ± 7.25	9.75(1.43–15.15)	–	–	0.475	–
FIB-4 score	5.00 ± 4.38	7.52 ± 4.32	5.23 ± 4.47	6.69(3.30–9.70)	6.56 ± 5.43	11.00 ± 7.74	0.999	** < 0.001**
Delta-FIB-4	1.50 ± 3.24	3.16 ± 3.54	1.60 ± 2.65	1.94(0.61–3.85)	–	–	0.212	–

The bold values were considered statistical significance

BMI—body mass index; ≥ F3—advanced fibrosis and compensatory liver cirrhosis; PEG ± RBV—pegylated interferons ± ribavirin; DAA—direct-acting antiviral; PR + DAA—pegylated interferons ± ribavirin + direct-acting antiviral; WBC—white blood cell; HGB—hemoglobin; ALT—alanine aminotransferase; AST—aspartate aminotransferase; GGT-γ—glutamy transpeptidase; Cr—creatinine; INR—international normalized ratio; AFP—alpha-fetoprotein; CTP—Child-Turcotte—Pugh; LSM—liver stiffness measurement; Delta LSM—the difference of LSM between before and after sustained virologic response; FIB-4—fibrosis-4 score; Delta FIB-4—the difference of FIB-4 between before and after sustained virologic response; *p*1 Value—the difference between baseline characteristics in derivation and internal validation; *p*2 Value—the difference between baseline characteristics in derivation and external validation

In the derivation cohort, genotyping revealed that 250 (64.94%) patients and 107 (27.79%) patients were infected with the 1b and 2a genotypes of HCV, respectively; HCV genotyping was not detected for the other 28 patients (7.27%). Of the 175 patients (45.45%) who received DAA therapy, 145 (37.92%) were treated with the PR regimen. Sixty-four patients (16.63%) were treated with PR + DAA. There were 322 patients (83.64%) who had ≥ F3 (advanced fibrosis and compensatory liver cirrhosis); 63 patients (16.36%) had decompensated liver cirrhosis. The median follow-up time was 61.00 (57.00–66.00) months, during which 37 patients (9.61%) developed HCC. The annual incidence of HCC was 1.73%. The cumulative incidences of HCC at 3, 5, and 7 years were 3.12%, 7.53%, and 9.61%, respectively. Table 1 shows that the HCC patients were more likely to be male (64.86%), to have alcohol use disorder, and to be treated with DAAs. Additionally, baseline white blood cell (WBC) count, hemoglobin (HGB) level, platelet count, and serum albumin levels were lower, but aspartate aminotransferase (AST) levels, bilirubin levels, international normalized ratio (INR), LSM, FIB-4, Child–Turcotte–Pugh (CTP) score, and delta- FIB-4 were higher (all *p* < 0.05).

In the internal cohort, 115 patients (69.28%) and 38 patients (22.89%) had HCV infections with the 1b and 2a genotypes, respectively; while HCV genotyping was not discovered in 13 individuals (7.83%). Of the 79 patients (47.59%) who received DAA therapy, 53 (31.93%) were treated with the PR regimen, and 34 patients (20%) were treated with PR + DAA. Among the enrolled patients, 149 (89.76%) were ≥ F3 patients and 17 (10.24%) were decompensated cirrhosis patients. Compared with the derivation cohort, the internal validation cohort had a higher proportion of patients with hyperlipidemia (20.48%, *p* = 0.020), and there were no significant differences in other indicators (all *p* > 0.05). In the external validation cohort, genotyping revealed that 1b (133 patients, 63.03%), 2a (46 patients, 21.80%), 3a (7 patients, 3.32%), 3b (9 patients, 4.26%) and 6a (15patients, 7.11%). A total of 157 patients (74.41%) received treatment with DAA, while 54 patients (25.59%) were treated with PR + DAA. Within the cohort, 179 patients (84.83%) were ≥ F3 patients and 32 (15.17%) patients were decompensated cirrhosis patients. The external validation cohort exhibited lower serum albumin levels, platelet count and higher bilirubin concentration, INR, CTP, and FIB-4 compared to the derived cohort.

The median follow-up periods for the internal and external validation groups were 60.50 (57.00–66.00) and 57.00 (50.00–62.00) months, respectively; the annual incidence of HCC was 1.78% and 4.09%, respectively. The HCC patients in the internal validation cohort had higher FIB-4 and delta-FIB-4 scores and lower baseline platelet count than did the non-HCC patients (all *p* < 0.05; Table 1). Age, HGB, platelet count, alanine aminotransferase (ALT) levels, serum albumin levels, bilirubin concentration, LSM, and FIB-4 score were different between the HCC patients and non-HCC patients in the external validation cohort (all *p* < 0.05; Table 1).

### Development and validation of the nomogram for HCC

**Fig. 2:**
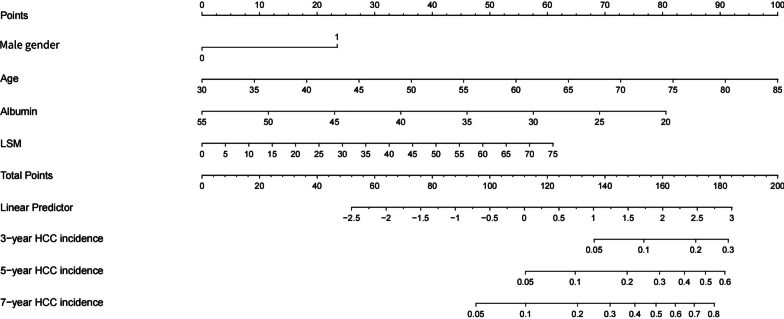
Nomogram for 3-, 5- and 7-year HCC incidence based on HCC risk point score

According to univariate Cox regression, the following indicators were significantly associated with development of HCC: age, sex, alcohol use disorder, DAA treatment, platelet count, serum albumin levels, bilirubin concentration, INR, LSM, delta-LSM, FIB-4 score and delta-FIB-4 score. Multivariate analysis revealed that older age (HR = 1.08, 95% CI 1.02–1.14, *p* = 0.009), male gender (HR = 2.38, 95% CI 1.10–5.13, *p* = 0.027), lower serum albumin levels (HR = 0.92, 95% CI 0.86–1.00, *p* = 0.037) and higher LSM (HR = 1.03, 95% CI 1.01–1.06, *p* = 0.001) were independent predictors of HCC development (Table 2). Thus, a nomogram was developed for evaluating the risk of hepatocellular carcinoma based on age, sex, serum albumin levels, and LSM (Fig. 2).

**Table 2:** Predictors of HCC by univariate and multivariate logistic regression analysis

Baseline variables	Univariate analysis			Multivariate analysis		
	Hazard ratio	95%CI	*p* Value	Hazard ratio	95%CI	*p* Value
Age, years	1.08	(1.02–1.14)	**0.006**	1.08	(1.02–1.14)	**0.009**
Male	2.46	(1.25–4.83)	**0.009**	2.38	(1.10–5.13)	**0.027**
BMI (kg/m^2^)	0.93	(0.85–1.02)	0.143			
Hypertension	0.56	(0.27–1.19)	0.132			
Diabetes	1.19	(0.57–2.45)	0.650			
Alcohol use disorder	2.65	(1.28–5.47)	**0.009**			
*Antiviral regimen*						
PEG ± RBV		Reference				
DAA	2.77	(1.24–6.20)	**0.013**			
PR + DAA	1.67	(0.58–4.81)	0.343			
Platelets (× 10^9^/L)	0.99	(0.99–1.00)	**0.004**			
ALT (IU/L)	1.00	(1.00–1.01)	0.662			
Albumin (g/L)	0.89	(0.84–0.93)	** < 0.001**	0.92	(0.86–1.00)	**0.037**
Bilirubin (µmol/L)	1.03	(1.02–1.05)	** < 0.001**			
INR	18.82	(4.78–74.29)	** < 0.001**			
Alpha-fetoprotein (ng/mL)	1.01	(0.99–1.02)	0.408			
LSM (kPa)	1.05	(1.03–1.07)	** < 0.001**	1.03	(1.01–1.06)	**0.001**
Delta-LSM (kPa)	1.04	(1.01–1.08)	**0.022**			
FIB-4 score	1.10	(1.06–1.15)	** < 0.001**			
Delta-FIB-4 score	1.14	(1.07–1.21)	** < 0.001**			

The bold values were considered statistical significance

*CI* confidence interval, *HR* hazards ratios

The Harrell's C-index of the derivation cohort was 0.81, indicating that the nomogram had good prediction accuracy. The nomogram was also found to be well calibrated based on a calibration plot (Additional file 1: Fig. S1), indicating that the predicted probabilities were reasonably close to the observed probabilities. The nomogram demonstrated satisfactory calibration and comparable prediction ability in both the internal and external validation cohorts (Harrell’s C indices were 0.78 and 0.73, respectively) (Additional file 1: Figs. S2, S3).

**Fig. 3:**
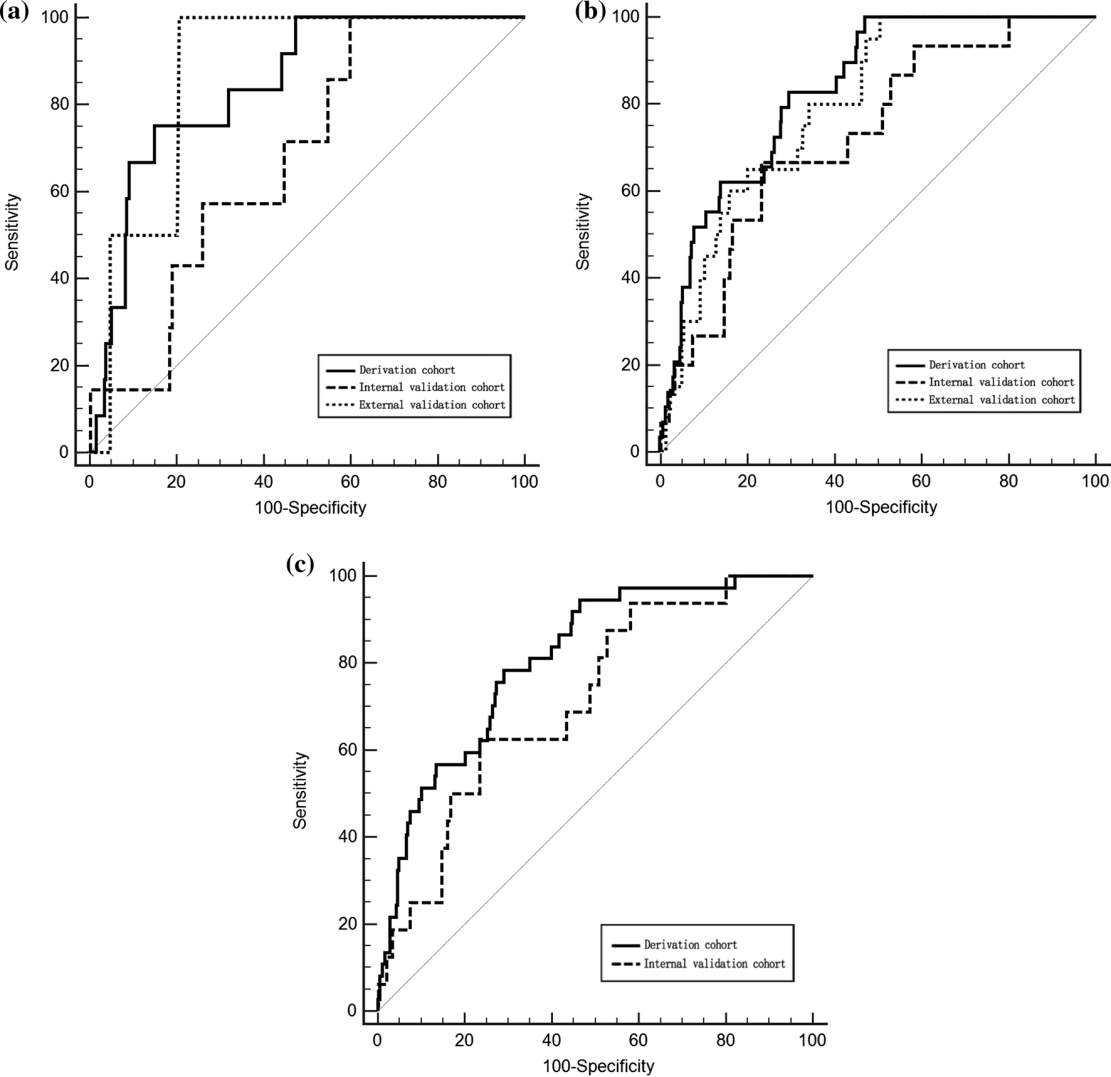
The time-dependent ROC of derivation cohort, internal validation cohort and external validation cohort

A time-dependent receiver operating characteristic (ROC) curve is essential for evaluating the accuracy of a nomogram, and the time-dependent ROC curve results showed similar prediction accuracy of the nomogram among the derivation, internal, and external validation cohorts. In the derivation cohort, internal and external validation cohort, the time-dependent AUROCS of the model at 3 and 5 years were as follows: 0.84 (95% CI 0.80–0.88) and 0.83 (95% CI 0.79–0.87); 0.68 (95% CI 0.61–0.75) and 0.73 (95% CI 0.66–0.80); and 0.87 (95% CI 0.82–0.92) and 0.80 (95% CI 0.74–0.85), respectively (multiple comparison, *p* > 0.05) (Fig. 3a, b). In the derivation and internal validation cohorts, the time-dependent AUROCS of the model at 7 years were 0.81 (95% CI 0.77–0.85) and 0.72 (95% CI 0.64–0.78), respectively (*p* > 0.05) (Fig. 3c).

### HCC cumulative incidence risk evaluated by the HCC risk score

**Fig. 4:**
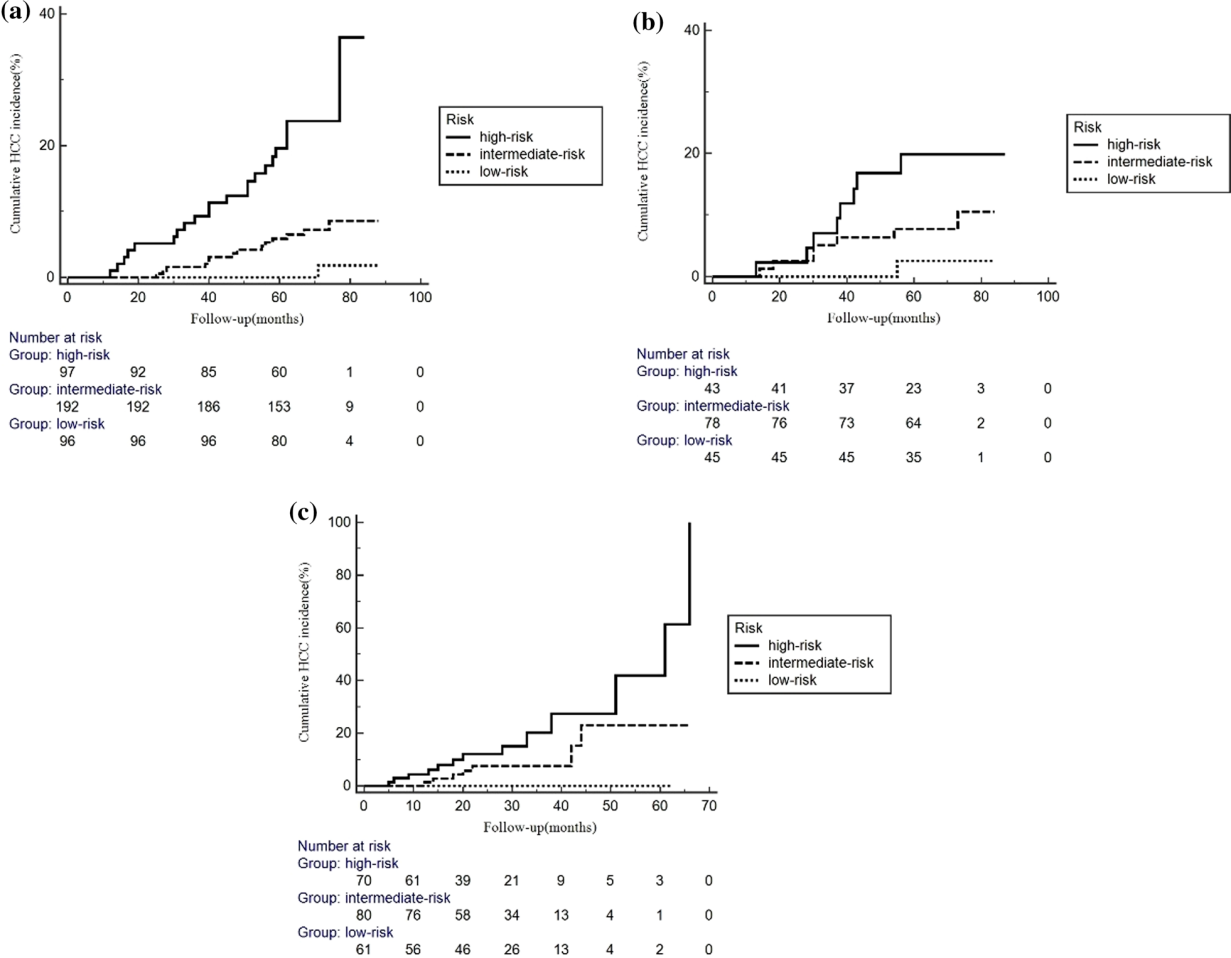
Cumulative HCC incidence in different risk groups. Patients were stratified by the 25th and 75th percentiles of the HCC risk score (< 95.45, 95.45–124.76, > 124.76 points)

Individualized risk assessment was performed with the established nomogram for HCC incidence according to the HCC risk score. The total of the relevant points for age, sex, serum albumin levels, and LSM was the HCC risk score. The patients were subsequently categorized based on the 25th and 75th percentiles of the HCC risk score (< 95.45, 95.45–124.76, > 124.76 points). In the low-risk (< 95.45 points), intermediate-risk (95.45–124.76 points), and high-risk (> 124.76 points) groups, the yearly incidences of HCC were 0.18%, 1.29%, and 4.45%, respectively (Table 3); the cumulative incidences were 0.00%, 1.56%, and 9.28% at three years, 0.00%, 5.73%, and 18.56% at five years, and 1.04%, 7.29%, and 22.68% at seven years, respectively (Table 3, Fig. 4).

**Table 3:** HCC incidence according to HCC risk score of derivation cohort

	Number of patients (N)	Incidence per-year (%)	3-Year cumulative incidence (%)	5-Year cumulative incidence (%)	7-Year cumulative incidence (%)
*Derivation cohort*					
Low-risk group	96	0.18	0.00	0.00	1.04
Intermediate-risk group	192	1.29	1.56	5.73	7.29
High-risk group	97	4.45	9.28	18.56	22.68
*Internal validation cohort*					
Low-risk group	45	0.39	0.00	2.22	2.22
Intermediate-risk group	78	1.60	5.13	7.69	8.97
High-risk group	43	3.80	6.98	18.60	18.60
*External validation cohort*					
Low-risk group	61	0.00	0.00	0.00	–
Intermediate-risk group	81	3.65	6.17	8.64	–
High-risk group	69	9.02	13.04	18.84	–

Low-risk group, HCC risk score < 95.45points; Intermediate-risk group, HCC risk score 95.45–124.76 points; High-risk group, HCC risk score > 124.76 points

In internal validation, the per-year HCC incidence was 0.39%, 1.60%, and 3.80% in the low-risk, intermediate-risk, and high-risk groups, respectively (Table 3); the annual incidence of HCC in the external validation cohort was 0.00%, 3.65%, and 9.02% respectively (Table 3). Table 3 and Fig. 4 display the cumulative occurrence of HCC in the internal and external validation cohorts.

### Comparison of predictive ability with a previous risk model [13]

One of the best models for predicting HCC in hepatitis C patients is the model series developed by Ioannou [13]. ‘Model of HCV’ is the name used herein. Four formulas for each of the four patient subgroups—cirrhosis/SVR, cirrhosis/no SVR, no cirrhosis/SVR, and no cirrhosis/no SVR—were included in the model. The primary predictive parameters in the models were four variables: age, platelet count, the ALT/AST ratio, and serum albumin levels.

**Fig. 5:**
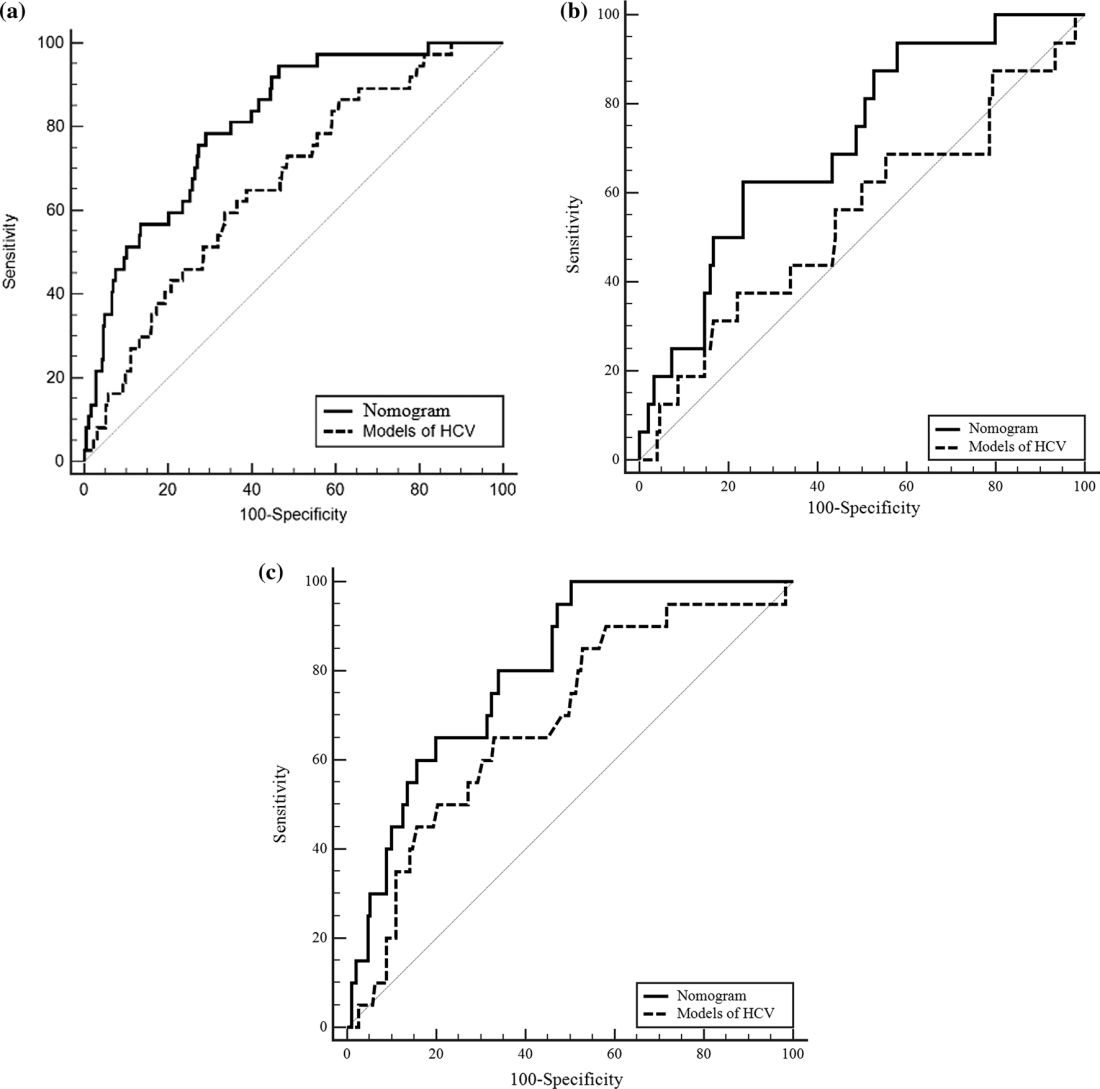
Comparison of prediction model

With respect to the derivation cohort and internal and external validation cohorts, the AUROCs of ‘Model of HCV’ were 0.66 (95% CI 0.62–0.71), 0.55 (95% CI 0.47–0.62), and 0.69 (95% CI 0.62–0.75), which were significantly lower than our prediction model (0.81 (95% CI 0.77–0.85), 0.72 (95% CI 0.64–0.78), and 0.80 (95% CI 0.74–0.85) (all *p* < 0.05) (Fig. 5).

## Discussion

HCC is one of the most common causes of liver-related mortality worldwide. According to the most recent guidelines from the AASLD and EASL [9, 11], people with chronic hepatitis C who have advanced fibrosis and cirrhosis need lifelong surveillance because they are still at risk of HCC even after SVR. These patients bear a severe burden owing to the screening strategy. According to current HCC guidelines, patients should be checked based on their risk of developing HCC. As there is no recognized risk model for these kinds of patients, we created and verified a unique nomogram that allowed us to categorize patients into low-, intermediate-, and high-risk groups, and a quarter of the individuals did not require lifetime screening because they were in the low-risk category.

The study's derivation cohort is unique. All the patients were living in the same county and became infected via plasmapheresis nearly simultaneously in the 1990s. For this reason, female patients accounted for 55.58%, and the genotypes of HCV were only 1b and 2a. The majority of the patients in the external validation cohort were city residents, and they were recruited at another central hospital in the city. Compared with those in the derivation cohort, the patients in the derivation cohort had lower serum albumin levels, lower platelet count, higher LSM, and higher FIB-4 scores, indicating that worse liver function, more severe fibrosis and greater portal hypertension. The derivation, internal, and external validation cohorts had annual HCC incidence rates of 1.73%, 1.78%, and 4.09%, respectively. The incidence ranged from 0.3% to 5.0%, which was consistent with previous data [13, 20, 21].

We developed a nomogram in this study using the following readily available indicators: age, male gender, serum albumin levels, and LSM. It is well known that male gender and older age are risk factors for HCC [13, 20, 22–24]. The serum albumin levels is a representative factor of liver function. It is commonly recognized that the increase in the incidence of HCC parallels the decrease in liver function [25]. Serum albumin levels was included in the aMAP and mPAGE-B HCC models [20, 23] as well as in our model.

As an accurate measurement of liver fibrosis, the LSM was also included in our model. Numerous studies have clearly demonstrated the beneficial association between LSM and HCC [16, 17, 26–36]. After SVR, the LSM decreases significantly as a result of fibrosis gradually regressing and inflammation progressing into remission [37, 38]. Recent research has shown a correlation between development of HCC and the baseline LSM as well as a reduction in the LSM. In an Italian cohort of 258 patients with HCV-related cirrhosis treated with DAA therapy [28], an LSM > 25–30 kPa was associated with a greater risk of HCC. Another Italian study [27] showed that the LSM at the end of treatment was reduced by more than 30% compared to baseline was associated with a significantly lower risk of HCC. Additionally, Alonso [15] verified that the 1-year delta-LSM was related to HCC incidence, and the baseline LSM and 1-year delta-LSM were both included in the established HCC prediction model (Harrell's C was 0.77). Similarly, our study demonstrated that the baseline LSM and delta-LSM were both independent risk factors for HCC; however, the baseline LSM was the more important component and was included in our model. We suggest that inclusion of the LSM is the reason why our model outperformed ‘Model of HCV’ [13] because the LSM outperformed the platelet count, AST/ALT ratio, and other indicators in evaluation of liver fibrosis. Although LSM should be measured by special equipment, FibroScan, has been used worldwide, including in China. We believe that our model will be suitable for most regions worldwide.

In the present study, platelet count [13, 22–24], FIB-4 score [17, 39] and delta-FIB-4 score [15, 17, 18, 39–41] were also found to be independent risk factors for HCC development, indicating that fibrosis is a determinant indicator. Several studies have demonstrated that fibrosis markers correlate with HCC incidence. For instance, a baseline FIB-4 score > 9 was linked to an increased risk of HCC in cohorts from Italy [41]. Alonso [15] constructed a prediction model with a baseline FIB-4 score and 1-year FIB-4 score (Harrell’s C was 0.81). Platelet count, FIB-4, and delta-FIB-4 may be useful for predicting HCC when FibroScan is not acceptable, even though they were not included in our model.

Current HCC guidelines suggest that patients be screened according to their risk of HCC. HCC incidence ≥ 1.5% per year has been found to be the most cost-effective screening threshold [42–44]. Recently, Farhang [45] demonstrated that patients with an HCC incidence lower than 0.5% per year need not undergo regular screening. Hence, there is an urgent need to accurately stratify HCC risk and refine current screening protocols. Our nomogram approach allowed us to categorize hepatitis C patients who achieved SVR with advanced fibrosis and cirrhosis into three categories based on their likelihood of developing HCC, low, intermediate, and high risk, and the three groups had annual incidences of HCC of 0.18% (24.86%), 1.29% (50.28%), and 4.45% (24.86%), respectively. In the internal and external validation cohorts, the incidences of HCC per year in the low-risk group were 0.00% and 0.39%, respectively (all *p* < 0.5%). Consequently, a quarter of patients might be spared from ongoing monitoring.

We have developed a model for individuals with advanced liver fibrosis and cirrhosis who have acquired SVR. From a methodological perspective, we validated the models using both external and internal cohorts and compared them to previous models. The objective and readily obtainable clinical variables that we incorporated in our model were age, male gender, serum albumin levels, and the LSM. This study's prediction model can also be applied with convenience in a clinical context. We also found certain limitations in our study. First, the LSM was one of the risk factors in our model. Although adding the LSM may increase the model’s capacity for prediction, it may also restrict its broad use. Second, the patients in the derivation group had low diversity. Although the model was further validated in a cohort from another city, its usefulness should be further validated in the future.

## Conclusion

Older age, male gender, low serum albumin levels, and high LSM were found to be risk factors for HCC after SVR in hepatitis C patients with advanced fibrosis and cirrhosis. Based on these risk factors, we established a nomogram for hepatitis C patients who achieve SVR and have advanced fibrosis or cirrhosis. Individuals who have a low risk of HCC, as determined by the model, may not require continuous monitoring. Our model was intended to lessen the burden on patients and improve the present HCC screening strategy.

### Supplementary Information

**Additional file 1. Fig. S1**. Calibration curves of derivation cohort. **Fig. S2**. Calibration curves of internal validation cohort. **Fig. S3**. Calibration curves of external validation cohort.

## Data Availability

The data that support the findings of this study are available upon request from the corresponding author. The data are not publicly available due to privacy or ethical restrictions.
