# Supplementary material for: Development and validation of a nomogram for assessing hepatocellular carcinoma risk after SVR in hepatitis C patients with advanced fibrosis and cirrhosis
Source: Infect Agent Cancer. 2024 Apr 25;19:17. doi: 10.1186/s13027-024-00578-3 (PMC11046761; doi:10.1186/s13027-024-00578-3)
Supplement: Supplementary file 1 — Additional file 1. Fig. S1. Calibration curves of derivation cohort. Fig. S2. Calibration curves of internal validation cohort. Fig. S3. Calibration curves of external validation cohort. [file 13027_2024_578_MOESM1_ESM.docx]

| 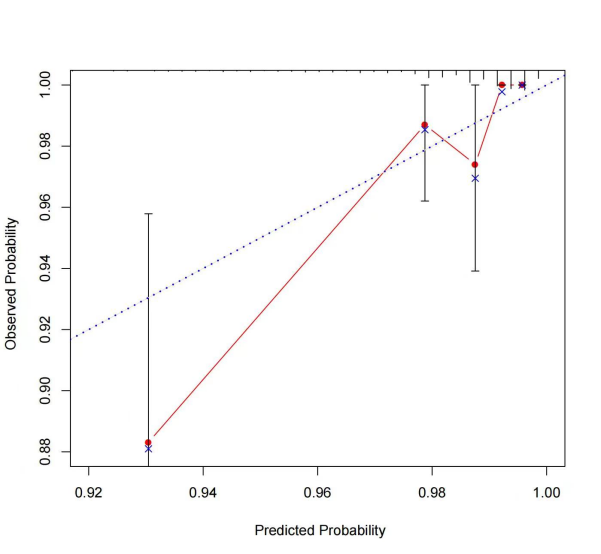  a: The graph represented the relationship between observed and predicted 3-year probability of HCC. | 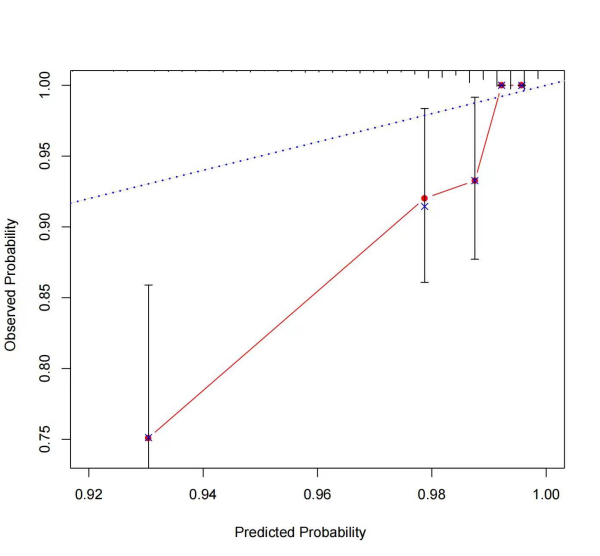  b: The graph represented the relationship between observed and predicted 5-year probability of HCC. | 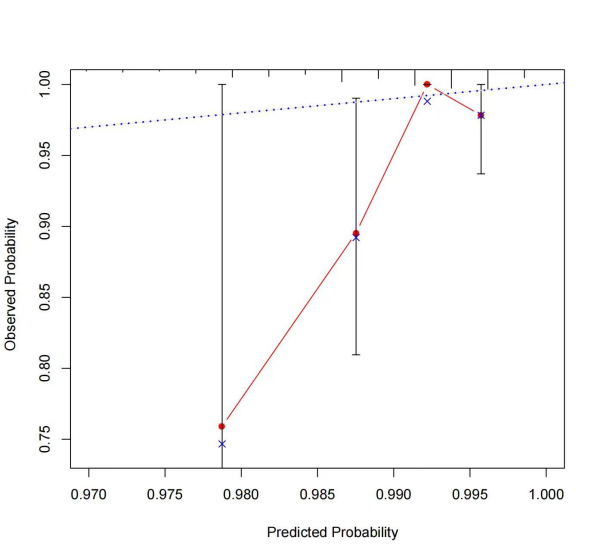  c: The graph represented the relationship between observed and predicted 7-year probability of HCC. |
| --- | --- | --- |

Fig. S1 Calibration curves of derivation cohort.

| 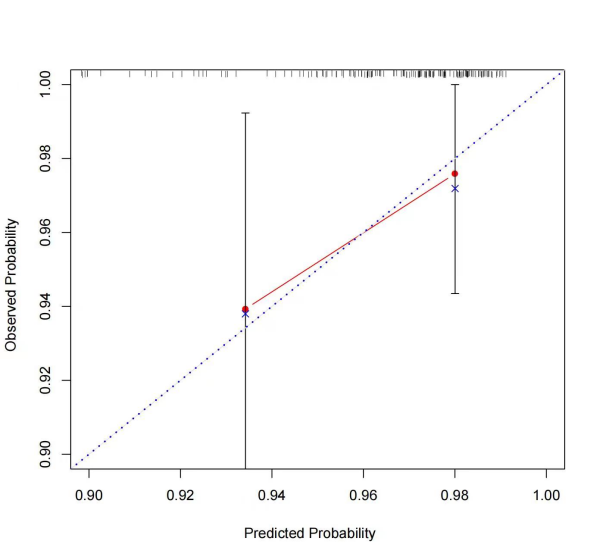  a: The graph represented the relationship between observed and predicted 3-year probability of HCC. | 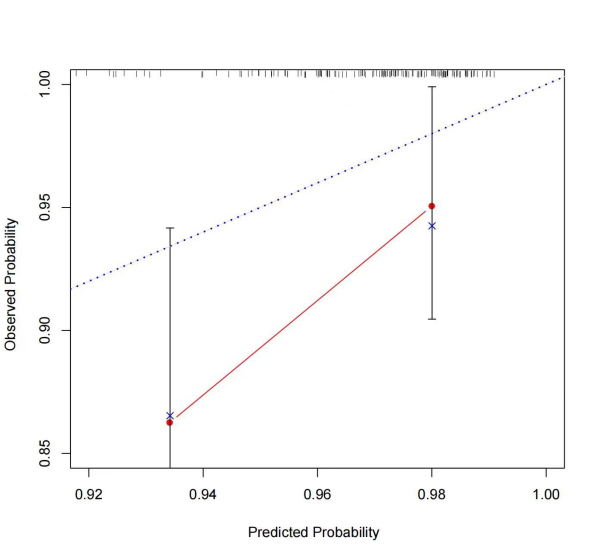  b: The graph represented the relationship between observed and predicted 5-year probability of HCC. | 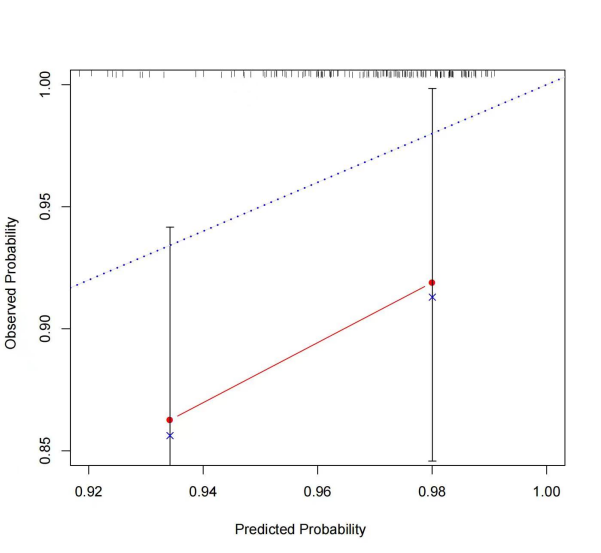  c: The graph represented the relationship between observed and predicted 7-year probability of HCC. |
| --- | --- | --- |

Fig. S2 Calibration curves of internal validation cohort.

| 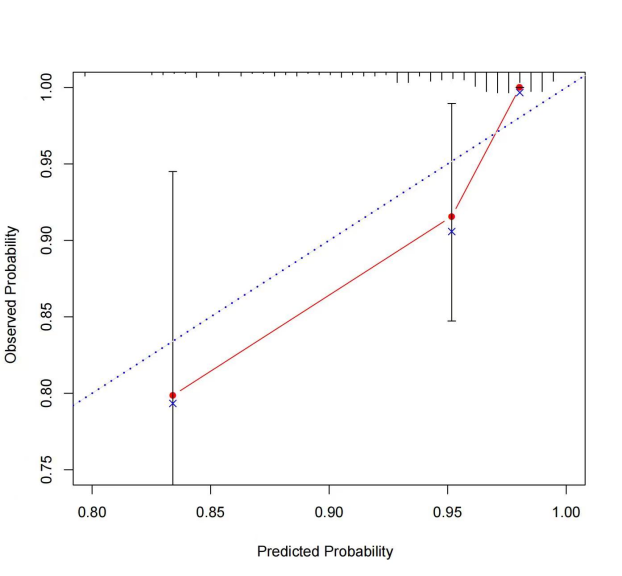  a: The graph represented the relationship between observed and predicted 3-year probability of HCC. | 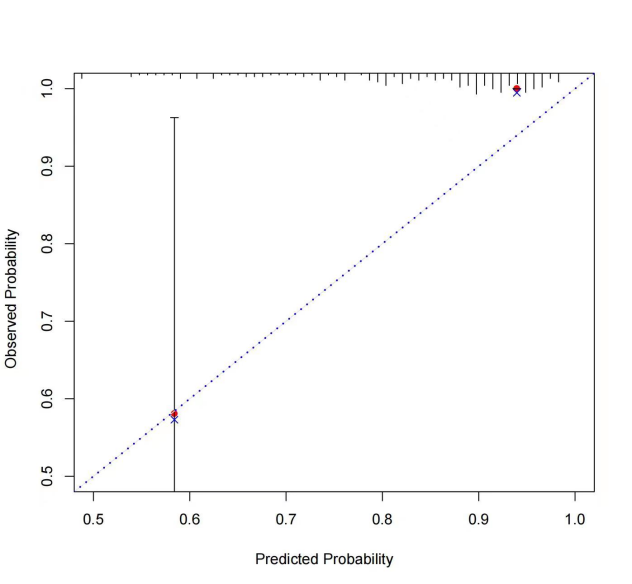  b: The graph represented the relationship between observed and predicted 5-year probability of HCC. |
| --- | --- |

Fig. S3 Calibration curves of external validation cohort.
